# Quaternion-Based Discriminant Analysis Method for Color Face Recognition

**DOI:** 10.1371/journal.pone.0043493

**Published:** 2012-08-24

**Authors:** Yong Xu

**Affiliations:** 1 Bio-computing Research Center, Shenzhen Graduate School, Harbin Institute of Technology, Shenzhen, Guangdong, People's Republic of China; 2 Key Laboratory of Network Oriented Intelligent Computation, Shenzhen, China; Université de Nantes, France

## Abstract

Pattern recognition techniques have been used to automatically recognize the objects, personal identities, predict the function of protein, the category of the cancer, identify lesion, perform product inspection, and so on. In this paper we propose a novel quaternion-based discriminant method. This method represents and classifies color images in a simple and mathematically tractable way. The proposed method is suitable for a large variety of real-world applications such as color face recognition and classification of the ground target shown in multispectrum remote images. This method first uses the quaternion number to denote the pixel in the color image and exploits a quaternion vector to represent the color image. This method then uses the linear discriminant analysis algorithm to transform the quaternion vector into a lower-dimensional quaternion vector and classifies it in this space. The experimental results show that the proposed method can obtain a very high accuracy for color face recognition.

## Introduction

Color images can provide a large quantity of appearance information of the real-world objects and allow the objects to be more accurately described than the grey-scale image [1]–[3]. In the field of face recognition, many literatures have shown that color face recognition usually can obtain a higher accuracy than conventional face recognition using the gray image of the face. There are three kinds of color face recognition methods. The first kind usually first converts the 3-D color space into a new lower-dimensional space and then perform classification in the new space. For example, an optimum conversion is proposed by Neagoe to transform the 3-D color space into a 2-D color space [4]. It was showed that the obtained 2-D color space was better for face recognition. Jones and Abbott proposed to convert the original 3-D color space to 1-D space, using Karhunen-Loeve (KL) analysis, linear regression, and genetic algorithms [5]. Yang et al. proposed the optimal discriminant model of color face images [6]. The second kind focuses on transforming the original color space into a new color space for better classification result. For example, Kittler and Sadeghi proposed the IG(R-G) color space for face verification [7]. This color space includes the following three color channels: an intensity (the mean of R, G, B channels), a chromaticity (normalized G) and an opponent chromaticity (normalized (R-G)) channel. Shih and Liu proposed the optimal color configuration 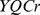 for color face recognition, where 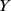 and 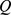 color components are from the 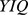 color space and 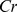 is from the 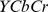 color space [8]. Liu proposed the so-called uncorrelated color space (UCS), the independent color space (ICS), and the discriminating color space (DCS) for color face recognition [9]. By using these spaces, a very high face recognition accuracy can be obtained [9]. Wang et al. used a sparse tensor discriminant color space (STDCS) model to represent the color image as a third-order tensor [10]. This model is able to preserve the underlying spatial structure of color images and to enhance robustness. The third kind integrates color information and the texture information for face recognition. For instance, Liu et al. used a hybrid color and frequency feature (CFF) method to perform color face recognition [11]. Liu et al. also fused multiple global and local features derived from a hybrid color space 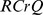 [12]. Choi et al. proposed color local Gabor wavelets (CLGWs) and color local binary pattern (CLBP) for face recognition [13]. The color local texture features proposed in [13] can use the discriminative information derived from spatiochromatic texture patterns of different spectral channels.

Color images require more storage space than grey-scale images. Moreover, the transmission of the color image also needs a larger bandwidth. The amount of the color image data such as a RGB, HIS, or YCbCr color image is usually three times of that of a grey-scale image with the same size. As a result, it is crucial to seek a way to effectively represent the color image in a low-dimensional space.

We note that classical image processing algorithm is not able to simultaneously mathematically deal with the three channels of the color image. Instead, when dealing with the color image, previous methods first separate the color image into three channels and then apply the traditional image processing algorithms to these three channels, respectively.

The quaternion can be used as a way to represent the color pixel consisting of three components [14]–[20]. Actually, the quaternion allows the three components of a color pixel to be simply denoted by a denotation in the form of a “number”. Moreover, the color image can be straightly represented by a quaternion matrix. Because a quaternion is composed of four real numbers, a simple means to represent the color pixel by a quaternion is to let three real numbers in the quaternion be respectively equal to the three color components of the color pixel and let the remaining real number in the quaternion be zero.

The quaternion representation of the color has been used in the context of color texture region segmentation [17]. Shi et al. also used a Hessian matrix defined on the basis of the quaternion to measure the curvature in color images [17]. Angulo [10] exploited the structure tensor of color quaternion image representations to perform feature extraction. Besides the color image, the quaternion was also used for greyscale images [10]. Moreover, we can also use a quaternion matrix to represent the multi-spectrum images such as three or four channel remote images.

A concise survey on matrices of quaternion entries is presented in [14]. Denis [16] studied the quaternionic Fourier spectrum and attempted to explain the color information contained in the new domain and how the different real and imaginary parts of the spectral quaternionic domain interact with the pure quaternion component chosen to encode colors in the spatial domain. Quaternion Fourier Transform was also proposed as a frequency analysis tool [20]–[23]. The quaternion has also been used to separate polarized waves and in block truncation coding [24], [25]. Miron et. al. [24] considered the problem of direction of arrival and polarization parameters estimation in the case of multiple polarized sources impinging on a vector-sensor array and devised a MUSIC-like algorithm to estimate direction of arrival of waves.

We also note that based on the quaternion algebra that was developed in recent years, we can also easily apply matrix operations to the quaternion matrix. This means that once we denote a color image by a quaternion matrix, we can directly exploit the quaternion algebra to implement some image processing techniques such as image denosing, image segmentation, edge detection and image transform [26]–[32].

In this paper, we aim at transforming the color image into lower-dimensional data for color face recognition. In order to do so, we extend the widely used linear discriminant analysis technique [33], [34] to the quaternion matrix that represents the color image. Previous research has shown that dimensional reduction is usually helpful for classification, as dimensional reduction is able to extract the important and robust features from the image and to neglect the trivial and non- salient information of the image. The experimental results show that the proposed method can not only use lower-dimensional quaternion array to describe the color image but also can achieve a high recognition accuracy. The proposed method has the following rationale: as it is a feature level fusion method, it can convey much richer information of the bimodal biometric traits than the matching score level fusion and decision level fusion methods [35]–[37]. The proposed method differs from the method proposed in [38] as follows: first, the proposed method and the method in [38] use a complex matrix and a real third-order tensor to denote the color face image, respectively. Second, as the proposed method and the method in [38] respectively perform calculation in a complex and real space, the three color components will be fused in two very different ways.

## Materials and Methods

### Denotations

A real quaternion, simply referred to as quaternion, is a vector
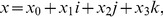
(1)where 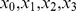 denote real coefficients. We note that the quaternion is defined in a four-dimensional vector space with an ordered basis, denoted by 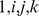. The conjugate of 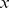 is defined by
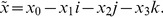
(2)Alternatively, we can also represent 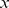 and 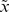 defined in (1) and (2) by 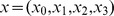 and 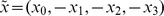, respectively. 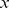 and 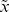 has the same norm 
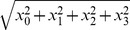
.

The product of any two of the quatemions 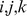 is defined by
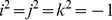
(3)

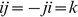
(4)

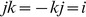
(5)

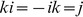
(6)


### Basis of the quaternion-based discriminant method

We first denote each color pixel of a color image by quaternion 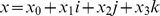 as follows: 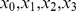 are set to 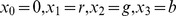, respectively. 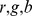 stand for the first, second and third components of the color pixel, respectively.

Suppose that there are 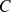 classes. Let 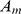 represent the quaternion matrix of the 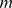
th color image, 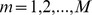. We can convert 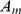 into a quaternion vector 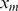 by concatenating the rows of 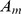 in sequence.

We define the generative matrix of the discriminant algorithm as
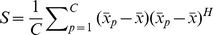
(7)where 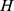 stands for conjugate transpose, 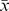 represents the mean of the quaternion vectors of all the training samples and 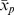 denotes the mean of the quaternion vectors of the training samples of the 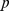
th class. 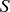 is indeed the covariance matrix of the class mean of the quaternion arrays corresponding to the training samples. The eigen-equation of quaternion matrix 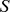 is as follows:
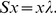
(8)


Once we obtain the eigenvalues and eigenvectors of 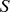, we can select the eigenvectors corresponding to the first 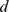 largest eigenvalues as the transform axes. We extract features from a sample represented by the quaternion array by respectively projecting this quaternion array onto the 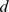 transform axes.

### Algorithm of the quaternion-based discriminant method

We note that as 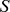 is a quaternion matrix, it is hard to directly solve its eigenvalues and eigenvectors. However, we can solve this problem in the following way: first of all we construct an equivalent complex matrix of 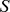. Then we solve the eigenvalues and eigenvectors of the equivalent complex matrix.

Corollary 1. If the quaternion matrix 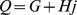, then the equivalent complex matrix 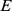 of 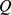 is defined as follows:
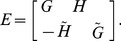
(9)If 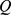 is a 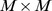 quaternion matrix, then 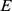 is an 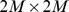 complex matrix.

For example, if 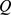 is the 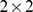 quaternion matrix 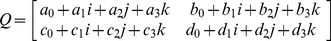, then we have 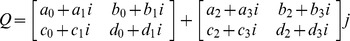. As a result, the equivalent complex matrix of 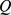 is the following 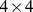 complex matrix:
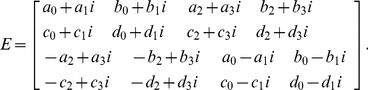
(10)


From the reference, we know real eigenvalues of 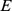 always appear as a pair and so do the complex eigenvectors of 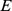. In other words, 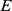 has the following eigenvalues and eigenvectors: 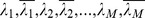; 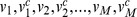. 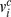 is the adjoint vector of 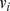, 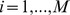. It is clear that 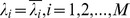. From the above context, we know that if 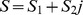, then 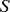 has the following equivalent complex matrix 
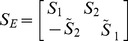
. We also note that eigenvalues and eigenvectors of the quaternion matrix and its equivalent complex matrix have the following relationship [14]: the complex eigenvalues of a quaternion matrix are the same as the eigenvalues of its equivalent complex matrix. In addition, if 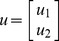 is the eigenvector with respect to the eigenvalue 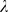 of equivalent complex matrix 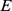, then 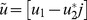 is also an eigenvector with respect to the eigenvalue 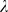 of quaternion matrix 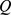. Hereafter 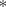 denotes the complex conjugate.

The main steps of the algorithm to implement the discriminant method are as follows.

Step 1. Represent each pix of every color image by a quaternion number and denote a color image by a quaternion vector.

Step 2. Use Eq.(7) to calculate the covariance matrix 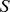 of the class mean of the quaternion arrays corresponding to the training samples. Then construct the equivalent complex matrix of the covariance matrix.

Step 3. Compute the eigenvalues 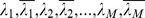 and eigenvectors 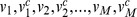 of the equivalent complex matrix. Suppose that the eigenvalues have the relationship 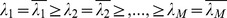. We exploit 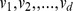 to construct a 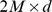 complex transform matrix 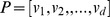.

Step 4. Convert the quaternion array corresponding to each color image into its equivalent complex matrix. For a quaternion array 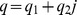, its equivalent complex matrix is 
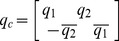
. Project 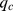 onto 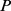 to generate the features 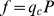.

Step 5. Exploit the nearest neighbor classifier to perform classification.

The method presented above is referred to as quaternion LDA.

## Results

In this section we use the Georgia Tech face database to test our method. Georgia Tech face database (GTFB) was built at Georgia Institute of Technology. GTFB contains images of 50 people taken in two or three sessions. All people in the database were represented by 15 color JPEG images with cluttered background taken at the resolution of 640×480 pixels. The pictures show frontal and/or tilted faces with different facial expressions, lighting conditions and scale. Each image was manually labeled to determine the position of the face in the image. We used the face images with the background removed.

**Figure 1:**
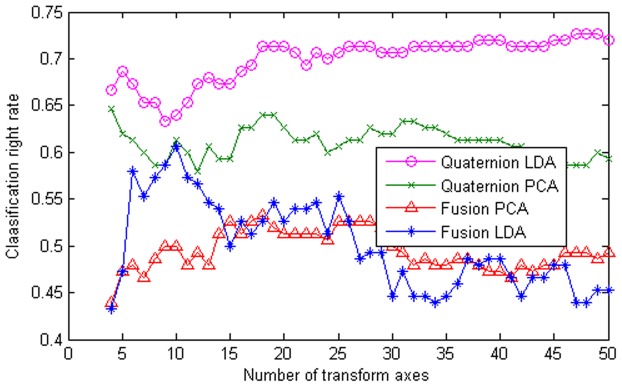
Classification accuracies of our method and several other methods on the GTFB database.

**Figure 2:**
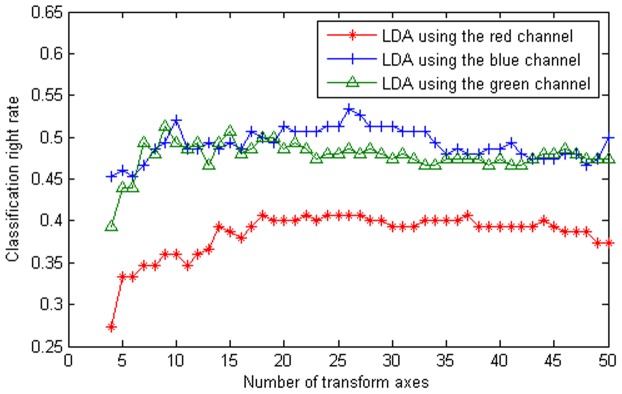
Classification accuracy of LDA using a single color channel on the GTFB database.

Since these images have different sizes, we first resized them into the same size of 40×30. We used the first 12 images of each subject as training samples and treated the remaining images as testing samples. Figure 1 shows the classification accuracies of our method and several other methods. Here ‘Fusion PCA’ represents the method that first performs PCA for the three channels of the color image and then uses the sum rule to fuse the matching scores of the PCA feature extraction results of the three channels for the ultimate face recognition. ‘Fusion LDA’ does similarly except that the performed feature extraction procedure is LDA rather than PCA. From Figure 2, we see that our method, quaternion LDA, obtains a much higher classification accuracy than quaternion PCA, fusion LDA and fusion PCA. Figure 2 shows classification accuracies of LDA using a single color channel on the GTFB database.

## Conclusion

This paper, for the first time, proposes the qauaternion-based discriminant analysis method. This method can represent the color images in a simple and tractable way. Since the proposed method is feature level fusion method, it is able to convey richer information of the color image than the score level and decision level fusion methods. Moreover, when the method transforms the color image into a very low-dimensional space, it can also well represent the image. The experiments show that the proposed method can perform very well in color face recognition.
